# Associations of Chronic Marijuana Use with Changes in Salivary Microbiome

**DOI:** 10.3390/microorganisms12112244

**Published:** 2024-11-06

**Authors:** Jun Panee, Yujia Qin, Youping Deng

**Affiliations:** 1 Department of Cell and Molecular Biology, John A Burns School of Medicine, University of Hawaii at Manoa, Honolulu, HI 96813, USA; 2 Department of Quantitative Health Sciences, John A Burns School of Medicine, University of Hawaii at Manoa, Honolulu, HI 96813, USA; yqin@hawaii.edu

**Keywords:** cannabis, smoking, microbiota, *Streptococcus vestibularis*, *Veillonella dispar*, *Prevotella melaninogenica*

## Abstract

The legalization of marijuana (MJ) for medicinal and recreational use has raised concerns about its potential impact on health, including oral health. While MJ use has been linked to poor oral health, its effects on the composition of the oral microbiome remain unclear. This cross-sectional study analyzed saliva samples from chronic MJ users (n = 18) and nonusers (n = 20) to investigate MJ-related changes in salivary microbiome composition. We identified significant differences in the relative abundance of 16 taxa, including seven species, such as *Megasphaera micronucliformis*, *Prevotella melaninogenica*, and *Streptococcus anginosus*. Additionally, five species showed positive correlations with cumulative lifetime MJ use, including *Streptococcus vestibularis* and *Streptococcus parasanguinis*. By grouping salivary microbial communities into clusters based on their association with periodontal health, we found that the cluster with species associated with poor periodontal health had the highest percentage of MJ users. Moreover, MJ use significantly contributed to variance in microbial communities in individuals with relatively good periodontal health. These findings suggest that chronic MJ use is associated with alterations in the salivary microbiome, highlighting its potential broader impact on oral and systemic health.

## 1. Introduction

Marijuana (MJ) usage has surged following its legalization for medical and recreational purposes in many Western countries. In the United States alone, nearly 50 million individuals reported MJ use within the past year, as per the National Survey on Drug Use and Health in 2020. Despite its widespread acceptance, understanding the implications of MJ use for human health, particularly oral health, remains crucial. Prior studies have associated MJ consumption with several oral health issues, such as diminished salivary flow, xerostomia (dry mouth), and heightened incidence of oral mucosal diseases and severe gingivitis, underlining potential concerns [1,2,3]. While emerging research has begun to illuminate the relationship between MJ use and the oral microbiome, for instance, an increase in pathogenic yeast *Candida albicans* prevalence following MJ smoking [4], and the identification of the cannabis use-associated oral microbiome species in cancer-prone oral mucosa sites [5] and saliva [6], a gap remains in fully elucidating this link.

Studies by Yamashita and Takeshita [7] established a connection between periodontal health and specific salivary bacterial cohabitating groups at both genus and species levels, categorizing them into discernible clusters that correlate with oral health statuses. These findings suggest that the balance between beneficial and harmful oral bacteria is influenced by various lifestyle factors, including tobacco use, positing these microbial groups as potential oral health biomarkers.

Building on this background, our study is designed to investigate the effects of chronic MJ use on the composition of the oral microbiome. In this study, we hypothesized that chronic MJ use, with smoking as the main route of intake, is associated with changes in bacterial composition in saliva, and the overall direction of these changes is geared toward poor oral health. Utilizing unstimulated saliva samples collected from 19 chronic MJ users and 20 nonusers in a previous pilot cognitive study [8], we explored potential associations between MJ use and salivary microbiome compositions. Target 16S rRNA sequencing was used to identify oral microorganisms at all taxonomic levels, and compositional changes in the salivary microbiome were analyzed in relation to MJ use. The oral health of the participants was not examined in this study; instead, cohabitating bacterial groups identified by Yamashita and Takeshita [7] were used as indicators of periodontal health.

## 2. Materials and Methods

**Participant Recruitment**. As previously reported [8], all participants were 18 years and older and recruited in Honolulu, Hawaii, between February 2015 and May 2015. Chronic MJ users had used MJ at least 3 times per week for at least 3 years with smoking as the main route of MJ intake and had urine toxicology positive for tetrahydrocannabinol (THC) but negative for cocaine, amphetamines, barbiturates, opiates, and benzodiazepine. In contrast, nonusers reported fewer than ten lifetime MJ uses, with no use in the six months preceding the study and were screened negative for the same substances. Recreational alcohol and cigarette use were allowed for all subjects. A comprehensive medical evaluation was conducted for each participant, including physical and neuropsychiatric assessments and a detailed review of their substance use and medical history. The physical evaluation included measurements of vital signs; a head-to-toe assessment that covers cardiovascular, respiratory, gastrointestinal, neuromuscular, and skin and lymphatic systems; and basic blood tests. The neuropsychiatric evaluation included a mental status examination, standardized cognitive tests, psychiatric symptom scales, and functional and quality of life assessments. We excluded participants with conditions that could confound study results, including major psychiatric illnesses, significant head trauma, severe chronic medical disorders, and current or history of other moderate to severe substance use disorders. Potential confounding factors such as age, sex, BMI, and MJ lifetime usage were also collected using questionnaires. All participants gave written informed consent, and the study’s protocol was sanctioned by the University of Hawaii’s Human Studies Program.

**Saliva Sample Collection and 16S rRNA Sequencing**. Unstimulated saliva samples were previously collected from all participants and stored in a −80 °C freezer. In January 2020, we sent the samples to the Genomics and Bioinformatics Shared Resource of the University of Hawaii Cancer Center (Honolulu, HI, USA), where DNA was extracted from these samples using the QIAamp DNA Mini kit (QIAGEN, Germantown, MD, USA), the V3–V4 regions of the 16S rRNA were sequenced using the Ilumina GAIIX sequencing platform (Illumina Inc., San Diego, CA, USA), and the amplicon libraries were generated according to Illumina TruSeq protocols.

**Microbiome Data Analysis**. The sequencing reads were pre-processed using the Illumina metagenomics workflow. Amplicon sequence variants (ASVs) present in at least 20% of the samples with more than 4 reads were retained, and the top 10% of ASVs with the least variance were removed to reduce noise. Alpha diversity indices were estimated using the R package “Vegan” (R version 4.3.1) [9]. Pearson correlation and linear regression analyses were conducted to assess relationships between taxa relative abundance, alpha diversity indices, MJ use, and other demographic factors. Beta diversity was measured using abundance-based Bray–Curtis distance and visualized through principal component analysis (PCA) plots. Microbial community clustering was based on eight indicative cohabitating species linked to oral health status [7]. These species were originally identified from the salivary microbiota of 2343 Japanese adults using next-generation sequencing, followed by a BLAST search against oral bacterial 16S rRNA gene sequences in the Human Oral Microbiome Database. Clustering was performed based on the relative abundances of predominant OTUs [10]. In our study, we expanded the clustering analysis to ASV, species, and genus levels, using clustered heatmaps to confirm the consistency and reliability of the clusters. To determine differences in the oral microbial community between MJ users and nonusers, alpha diversity was tested using Student’s *t*-test, while the relative abundance of individual taxa was analyzed using the Kruskal–Wallis test. Differences in the distribution of MJ users and nonusers across clusters were assessed with the chi-square test. Additionally, we used non-parametric dissimilarity tests, including ANOSIM (analysis of similarities), ADONIS (PERMANOVA), and MRPP (multiple response permutation procedure), to evaluate community similarities among different groups, such as sex, race, MJ use, and clusters.

This study was conducted in accordance with the STROBE guidelines (v4) for cohort, case–control, and cross-sectional studies.

## 3. Results

### 3.1. Characteristics of Study Participants

Out of the 39 participants, one MJ user was excluded from downstream data analysis due to DNA sequencing failure. The characteristics of the remaining 38 participants are listed in Table 1. Overall, the MJ users and nonusers had matched age, sex, race, ethnicity, education, and BMI. Compared to nonusers, MJ users started tobacco use 13.2 years earlier in life (*p* = 0.003) and showed a trend of having used tobacco for a longer duration (*p* = 0.055). For alcohol use, MJ users had marginally (0.05 < *p* < 0.1) younger age for first use, a higher amount of daily use, and more of them used alcohol in the past month compared to nonusers.

### 3.2. Composition and Diversity of the Salivary Microbiome

**Figure 1:**
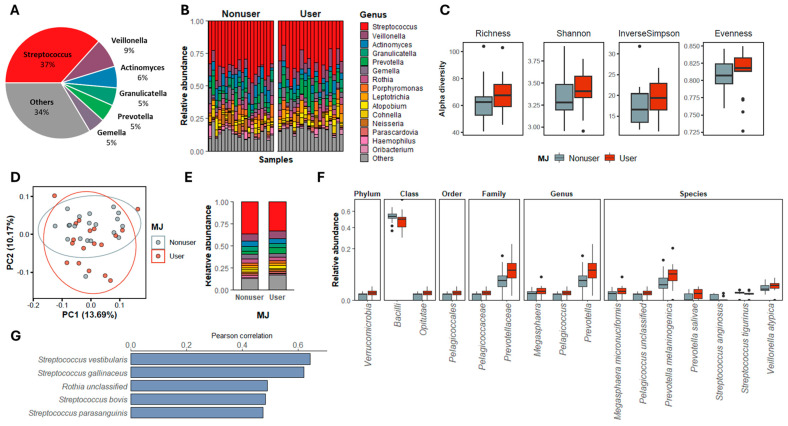
Saliva microbiome compositions at genus level and the characteristic comparisons between MJ users and nonusers. (**A**) Top six abundant genera identified in all the saliva samples in this study. (**B**) The microbial community composition in both MJ user and nonuser samples at the genus level; only the 15 most abundant genera are shown. (**C**) Alpha diversity indices, including species richness, Shannon index, and inverse Simpson and Pielou’s evenness in MJ user and nonuser groups. (**D**) Principal component analysis (PCA) plot showing the beta-diversities of MJ users and nonusers’ salivary microbial communities. (**E**) Average relative abundance of the 15 most abundant genera from MJ users and nonusers. (**F**) The taxa at all taxonomic levels that showed significantly different (*p* < 0.05, Kruskal–Wallis test) relative abundance between MJ users and nonusers. (**G**) The salivary species that showed significant correlations with MJ lifetime use (g) in the MJ user groups.

After excluding low-quality and non-bacterial sequences, the sequences were classified at different taxonomic levels (Figure 1A,B, Figure S1 in Supplementary Materials), and 99.18% of the reads can be classified at the genus level while 55.30% can be classified at the species level (Figure S1 in Supplementary Materials). In total, 81 out of the 182 ASVs were qualified for downstream data analysis, which accounts for 97.34% of the total sequence reads. These ASVs belong to 51 species and 37 genera, and among these, *Streptococcus* is the most abundant genus identified, while *Streptococcus vestibularis* is the most abundant species.

Alpha diversity analysis revealed no significant differences between MJ users and nonusers or across sexes and races (Figure 1C, Figure S2A,B in Supplementary Materials). We correlated the α-diversity indices with age, BMI, and lifetime MJ usage (Figure S2D in Supplementary Materials), and found a trend of positive correlation between Pielou’s evenness and BMI (*p* = 0.073) and a positive correlation between age and BMI (*p* = 0.026). Furthermore, beta diversity assessments using Bray–Curtis distances indicated no distinct clustering of microbial communities by MJ use, sex, or race (Figure 1D, Figure S2C in Supplementary Materials), which were also confirmed by the dissimilarity tests (Table S1, top row).

### 3.3. Bacterial Taxa Associated with MJ Use

To identify the salivary bacterial taxa that are associated with MJ use, we compared the relative abundances of all taxa between MJ users and nonusers using Kruskal–Wallis tests (Figure 1E). Among the 16 taxa (all taxonomic levels) that showed differential relative abundances based on MJ use (Figure 1F), 13 had higher abundances in MJ users than in nonusers, and these included one cohabiting genus and two cohabiting species that are associated with poor periodontal health. To evaluate the potential influences of MJ use on salivary microbiome composition at the species level, we assessed the relationship between species relative abundance and lifetime MJ usage among MJ users using Pearson correlation (Figure 1G, Figure S2E in Supplementary Materials). Notably, *S. parasanguinis* is a cohabitating species associated with poor periodontal health, as reported in [7].

### 3.4. Salivary Microbial Community Clustering and MJ Status in Oral Health

**Figure 2:**
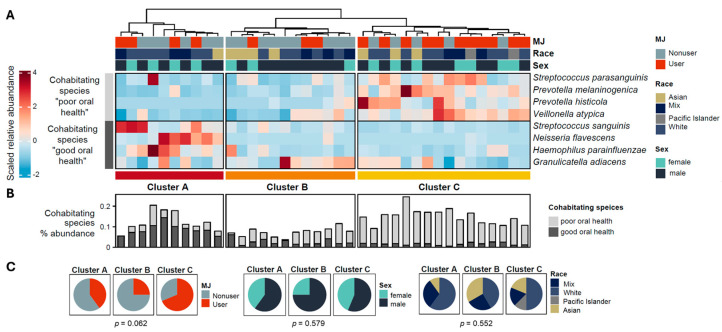
Microbial community clustering based on the salivary cohabitating species indicating the oral health conditions. (**A**) Heatmap shows three clusters (Clusters A, B, and C) were categorized based on the relative abundance of the eight salivary cohabitating species, which can be associated with good or poor oral health. (**B**) Distribution of cohabitating species associated with good or poor oral health among the three clusters. (**C**) Distribution of marijuana (MJ) users and nonusers, sex, and race among the three clusters. *p*-values were calculated using the chi-square test.

To explore the association between MJ use and oral health, we applied the framework of Yamashita and Takeshita (2017) [7], categorizing the salivary microbiome into cohabitating groups based on the abundance of key species linked to periodontal health, with 9 of the 11 identified species replicated in our study. Except for *Veillonella parvula* (Figure S3 in Supplementary Materials), the other eight species distinctly clustered into two cohabitating groups correlating with periodontal health status (Figure 2A). Based on the relative abundance of these eight species, three clusters (namely Clusters A, B, and C) can be yielded from the 38 salivary microbial communities using hierarchical clustering (Figure 2A). Among these three clusters, Cluster A showed a high abundance of species indicative of good oral health, while Cluster C was enriched with species associated with poor health (Figure 2B). Interestingly, we noted a marginal discrepancy in MJ status among the clusters (*p* = 0.062), with Cluster A showing a much lower percentage of MJ users compared to Cluster C (Figure 2C).

**Figure 3:**
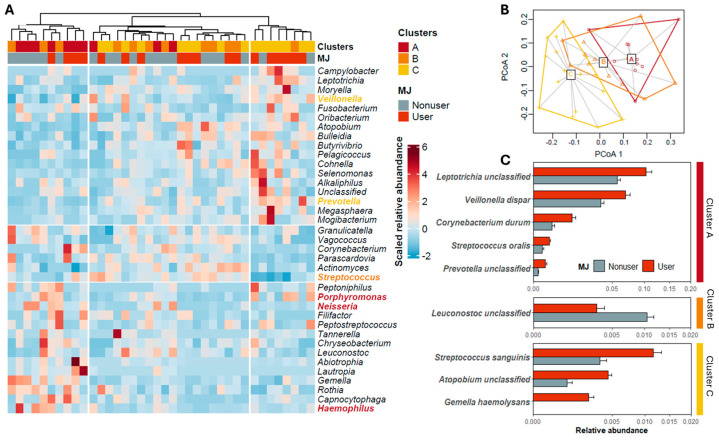
Validation and characteristics of the three defined clusters (Clusters A, B, and C) and MJ-associated species in each of them. (**A**) Genus-based clustering of microbial communities. Three clusters were formed based on the relative abundance of all 37 genera identified in this study, and they are highly correlated with the clusters identified in Figure 2. The genera with names labeled in colored text belong to the salivary cohabitating groups illustrated in Figure 2. (**B**) Dispersions of the microbial community in each cluster. (**C**) ASVs with significantly different relative abundance between marijuana (MJ) users and nonusers. The comparisons were made within each of the three clusters. *p* < 0.05, Kruskal–Wallis test.

To validate the reliability of the eight cohabitating species-based clustering, we further clustered the samples based on the relative abundance of the 37 genera identified in this study. This all-genus-based classification also resulted in three clusters (Figure 3A), exhibiting a strong correlation with the eight species-based clusters (*p* < 0.001, chi-square test). Notably, six of the seven cohabitating genera proposed by Yamashita and Takeshita were identified in our study and clustered precisely as proposed by the authors. Additionally, a clustered heatmap created using the 81 identified ASVs separated the 38 samples into two distinct groups, with all samples in Cluster A consistently grouping together (Figure S4A Supplementary Materials).

We then examined how MJ use might influence the microbiome within these clusters. While no significant alpha diversity differences were detected across clusters (Figure S4B in Supplementary Materials), the overall salivary microbiome structures were significantly different among clusters (*p* < 0.001, Table 2, Table S1 in Supplementary Materials). The PCA plot (Figure 3B) also showed that Cluster C was clearly separated from Clusters A and B, even though the variances within each cluster are similar (Figure S4C in Supplementary Materials). Table 2 presented the results of PERMANOVA analysis, revealing the factors contributing to intra-cluster ASV variance. Notably, MJ status was identified as a significant contributor (*p* = 0.045), explaining 16.21% of the variance in Cluster A but not in other clusters. In Cluster A, age and BMI exhibited marginal contributions to microbiome variance (0.05 < *p* < 0.1), with BMI emerging as the sole significant contributor to variance in Cluster C (*p* = 0.004).

We investigated microbiome changes associated with MJ use in each cluster, and found that specific bacteria like *Veillonella dispar*, *Corynebacterium durum*, and *Streptococcus oralis* in Cluster A, and *Streptococcus sanguinis* and *Gemella haemolysans* in Cluster C, were more abundant in MJ users compared to nonusers (Figure 3C). Although alpha diversity indices (Figure S4D in Supplementary Materials) did not reveal significant disparities between MJ users and nonusers within each cluster, PCA highlighted a clear distinction in Cluster A’s microbiome structure between MJ users and nonusers, suggesting a link between MJ use and microbiome composition, especially in individuals with better predicted oral health.

## 4. Discussion

### 4.1. Major Salivary Bacterial Species Associated with MJ Status and Their Potential Effects on Health

In this study, we found that three of the five most abundant salivary bacterial species (Figure S1E in Supplementary Materials) are significantly associated with marijuana (MJ) usage. Notably, *Streptococcus vestibularis*, constituting 7.38% of the microbiome, correlates positively with MJ use among MJ users. This correlation is particularly intriguing given the similar elevations observed in tobacco users [11]. While *S. vestibularis* has rarely been associated with human diseases, its variable abundance is associated with diverse health conditions, ranging from oral health (Bao et al. 2020 [12]) and viral infection (Tsang et al. 2020 [13]) to schizophrenia (Zhu et al. 2020 [14]) and chronic obstructive pulmonary disease (Bowerman et al. 2020 [15]), meriting further investigation into its role in MJ users.

Another abundant salivary species identified in this study is *Veillonella dispar*, which accounted for 4.46% of salivary microbiome and showed higher relative abundance among MJ users than nonusers in Cluster A. Similarly, *V. dispar* has also been found to be more prevalent in tobacco smokers. One of the most noticeable characteristics of *V. dispar* is that it produces nitrite by reducing nitrate [16] and may thus affect oral health from multiple aspects (Feng et al. 2023 [17]). *V. dispar* abundance in oral tissues varies with age, being more prevalent in healthy tissues of elderly adults [12,18,19] but associated with caries-active status in children [20,21], suggesting that MJ-related increases in *V. dispar* levels in saliva could affect oral health in an age-dependent manner.

*Prevotella melaninogenica*, the fourth most prevalent species, was also more abundant in MJ users. This species has also been reported to be more abundant in the saliva of tobacco smokers [22] and methamphetamine users [23] compared to control groups. *P. melaninogenica*’s production of pro-inflammatory lipopolysaccharide (LPS) renders it periodontopathic, contributing to inflammatory progression in oral lichen planus patients [24], caries in children [19], and active periodontal disease in the general population [25]. Additionally, its presence is linked to various cancers [26,27], high blood pressure [25], and increased risk of Alzheimer’s disease mortality [28], raising important questions about the broader health implications of its increased abundance in the context of MJ use.

### 4.2. Salivary Bacterial Species Associated with Both MJ and Tobacco Smoking

The most significant demographic difference between MJ users and nonusers in this study is that MJ users started tobacco use 13.2 years earlier in life (*p* = 0.003) than nonusers did, although the two groups reported similar accumulative lifetime use and recency of use. As previously discussed, tobacco smoking is associated with increased abundances of *S. vestibularis*, *V. dispar*, and *P. melaninogenica* in the mouth, which are the major salivary species affected by MJ use. Additionally, four more species identified in this study show similar patterns. *Megasphaera micronuciformis*, *Prevotella salivae*, and *Veillonella atypica* have higher salivary abundances among MJ users than nonusers; they are also more abundant in the buccal swab samples from tobacco smokers in comparison to non-smokers [11], and *M. micronuciformis* is also enriched in the upper gastrointestinal tract of tobacco smokers [29]. *Streptococcus sanguinis* is another salivary species that is more abundant among MJ users than nonusers within Cluster C, and its abundance also increases in the subgingival plaques of tobacco (shisha) smokers compared to non-smokers [30]. The observations can be explained from two perspectives: (1) Early tobacco use: Although the MJ users and nonusers had similar accumulative lifetime use and recency of use, MJ users began using tobacco at an earlier age. This earlier exposure to tobacco may significantly impact the composition of the salivary microbiome. (2) Impact of smoking on the oral environment: Both tobacco and MJ smoking alter the oral environment in ways that can affect the microbiome. These changes include exposure to heat, reduced saliva production (dry mouth), decreased blood flow to the gums, plaque and tartar buildup, and thickening and discoloration of oral soft tissues. Such alterations can substantially influence the microbiome composition. While both groups shared similar levels of lifetime tobacco exposure, only MJ users were exposed to MJ smoke, which may result in more pronounced changes in smoking-associated bacteria, as observed in this study.

### 4.3. Does MJ Use Affect Oral Health via Affecting the Structure of the Microbial Community?

Using Yamashita and Takeshita’s (2017 [7]) proposed cohabitating species, the salivary microbiomes in this study were grouped into three clusters. Cluster C, which included the highest proportion of MJ users, also exhibited increased levels of species linked to adverse periodontal health, suggesting a potential connection between MJ consumption and deteriorating oral health. Furthermore, MJ use was the only demographic factor tested in this study that explains the variance of the microbiomes in Cluster A (Table 2). This cluster was characterized by higher abundances of species associated with optimal periodontal health (Figure 2B), indicating that MJ usage could be linked to significant microbial shifts even in individuals with otherwise healthy oral environments. This finding suggests that MJ use might induce notable shifts in the oral microbiome, especially among individuals with generally healthy oral conditions, pointing to the importance of further investigating the role of MJ in oral microbial dynamics and health.

### 4.4. Comparative Analysis of Factors Influencing the Salivary Microbiome in MJ Users

Luo et al. reported in 2021 [6] that salivary microbiome changes were associated with chronic marijuana, with a sample size similar to ours. However, key distinctions exist between the cohorts of both studies. Firstly, our participants used MJ recreationally, averaging 1.2 g daily, whereas Luo et al.’s subjects [6], diagnosed with cannabis use disorder, consumed significantly higher THC doses. Secondly, in Luo’s study, some participants had ceased MJ use for over three weeks before sampling, unlike the consistent daily usage in our cohort. Lastly, while Luo et al. focused on Caucasian and African American participants, our study encompassed a broader racial diversity, though no African Americans were recruited. These differences could account for the disparate findings as follows: Luo et al. observed reduced β-diversity and significant changes in 36 species, contrasted with the 7 species identified in our study. The comparison between the two studies suggests that MJ dose, race, and duration of abstain may separately or interactively affect the composition of salivary microbiome.

### 4.5. Strength and Limitation of This Study

This research stands out as one of the pioneering studies to extensively examine the salivary microbiome in MJ users compared to nonusers, identifying distinct group differences across various taxonomic levels. It is notably the first to apply the concept of salivary cohabitating groups, as introduced by Yamashita and Takeshita [7], yielding significant insights into the microbial community structure at both the genus and species/ASV levels. Despite these contributions, the study faces certain limitations. The sample size represents the maximum feasible recruitment we could achieve within the given study period and with the resources available. While we acknowledge that this number is smaller than ideal for generalizing findings broadly, it nonetheless provides valuable insights into the effects of chronic MJ use on the salivary microbiome and lays the groundwork for future research in this area. The lack of direct oral health assessments limits our ability to establish a definitive connection between MJ use and oral health outcomes. To compensate for this limitation, we used the cohabitating groups as a surrogate periodontal health indicator. Additionally, the absence of dietary data prevents us from assessing the potential impact of diet on the microbiome differences observed. Moreover, the interplay between earlier and more prolonged tobacco use among MJ users and its influence on the microbiome composition requires further exploration to fully understand the confounding effects of tobacco.

## 5. Conclusions

This study shows that chronic MJ use via smoking is associated with significant changes in relative abundances of 16 taxa across all taxonomic levels, and lifetime MJ use dose-dependently correlates with relative abundances of five species in the salivary microbiome. Several of these MJ-affected taxa belong to the cohabitating groups associated with poor periodontal health. MJ use significantly contributes to changes in salivary microbiome composition among people with relatively good periodontal health, as implicated by the cohabitating groups. Future studies should include oral health evaluation of the participants and samples from multiple locations of the oral cavity for a more comprehensive understanding of the effects of MJ use on oral microbiome composition and oral health outcomes.

## Figures and Tables

**Table 1:** Participant characteristics. Data are shown in number, %, or median (interquartile range).

	Marijuana Users (n = 18)	Nonusers (n = 20)	*p* Value ^1^
Age (years)	27.5 (22.3–31.5)	27.0 (21.5–36.0)	0.91
Sex (female/male)	7/11	7/13	0.90
% Race (Asian/mixed/Pacific Islander/White)	11.1/27.8/11.1/50	30/20/0/50	0.26
% Ethnicity (Hispanic/non-Hispanic)	27.8/72.2	15/85	0.44
Education (years)	14.5 (12.3–16)	14.8 (14–16.3)	0.25
Body Mass Index (kg/m^2^)	23.5 (21.7–27.6)	26.3 (23.6–29.6)	0.45
Waist/hip circumference ratio	0.86 (0.83–0.88)	0.86 (0.82–0.92)	0.93
Neck circumference (cm)	36.3 (33.6–38.0)	36.5 (34.5–39.0)	0.65
***Substance Use Patterns***			
**Marijuana (MJ) Use**			
# used MJ in past month (%)	18/18 (100%)		
Age of first MJ use (year)	16.0 (15.0–18.8)		
Daily average MJ used (g)	1.2 (0.6–2.0)		
Duration of MJ use (year)	7.3 (4.0–10.8)		
Total lifetime MJ used (kg)	2.7 (1.6–4.7)		
**Alcohol Use**			
# used alcohol in past month (%)	16/18 (90%)	12/20 (60%)	0.064
Age of first alcohol use (year)	19.0 (16.0–22.0)	21.0 (20.0–21.0)	0.057
Daily average alcohol used (mL)	6.7 (2.7–12.1)	1.6 (0.5–7.3)	0.066
Duration of alcohol use (year)	8.5 (4.25–10.75)	2.0 (1.0–8.0)	0.10
Alcohol use abstinence (day)	4.0 (1.0–9.0)	14.0 (4.0–30.0)	0.14
Lifetime alcohol used (L)	19.4 (9.8–28.0)	0.6 (0.2–19.0)	0.10
**Tobacco Use**			
# used tobacco in past month (%)	5/18 (28%)	3/20 (15%)	0.44
Age of first tobacco use (year)	15.8 (14.5–17.9)	29.0 (26.0–36.0)	**0.003**
Daily tobacco use (cigarette#/day)	0 (0–5.0)	0 (0–0.8)	0.99
Duration of tobacco use (year)	0 (0–11.9)	0 (0–0.5)	0.055
Nicotine abstinence (day)	3.5 (0–489.5)	11.0 (0–62.0)	0.98
Lifetime nicotine used (g)	0 (0–157.2)	0 (0–19.6)	0.58

^1^ *p*-values were calculated using chi-square or *t*-tests, as appropriate. *p*-values < 0.05 are highlighted in bold.

**Table 2:** Salivary microbiome variance explained by marijuana (MJ) use, sex, BMI, and age. Study participants (n = 38) were clustered into A, B, and C based on two cohabitating species groups; all 81 amplicon sequence variants (ASVs) identified in this study were used for the PERMANOVA test.

	All		Cluster A		Cluster B		Cluster C	
	R^2^	*p*	R^2^	*p*	R^2^	*p*	R^2^	*p*
**Cluster**	**20.84%**	0.001 *	**-**	**-**	**-**	**-**	**-**	**-**
**MJ**	3.78%	0.154	**16.21%**	0.045 *	5.04%	0.92	3.76%	0.898
**Sex**	3.21%	0.292	6.98%	0.929	10.01%	0.271	5.98%	0.509
**BMI**	2.69%	0.401	15.92%	0.09	8.58%	0.448	**17.70%**	0.004 *
**Age**	2.99%	0.31	16.51%	0.078	12.19%	0.186	8.43%	0.207

R^2^: variance of the microbiomes explained by the factors. * *p* < 0.05, PERMANOVA test.

## Data Availability

The raw sequencing data and supplementary information have been securely deposited into the NCBI Sequence Read Archive (SRA) and are now openly accessible under the accession number PRJNA995421.

## Supplementary Materials

The following supporting information can be downloaded at: https://www.mdpi.com/article/10.3390/microorganisms12112244/s1, Figure S1: Overview of salivary microbiome composition at different taxonomic levels; Figure S2: Salivary microbiome structures based on other demographic factors and species associated with lifetime MJ usage; Figure S3: Clustering based on 9 salivary cohabitating species associated with good or poor oral health; Figure S4: Microbial community analysis based on the three clusters (Clusters A, B, and C) identified in Figure 2; Table S1: Dissimilarity tests of saliva microbial community structure based on all 81 amplicon sequence variants (ASVs) identified in this study.
