# Supplementary material for: Associations of Chronic Marijuana Use with Changes in Salivary Microbiome
Source: Microorganisms. 2024 Nov 6;12(11):2244. doi: 10.3390/microorganisms12112244 (PMC11596347; doi:10.3390/microorganisms12112244)
Supplement: Supplementary file 1 [file microorganisms-12-02244-s001.zip › microorganisms-3293424-supplementary.pdf]

Supplemental Materials

Associations of Chronic Marijuana Use with Changes in Salivary Microbiome

Jun Panee [1,*,†], Yujia Qin [2,†] and Youping Deng [2,*]

[1] Department of Cell and Molecular Biology, John A Burns School of Medicine, University of Hawaii at Manoa, Honolulu, HI 96813, USA

[2] Department of Quantitative Health Sciences, John A Burns School of Medicine, University of Hawaii at Manoa, Honolulu HI 96813 USA

[*] Correspondence: junchen@hawaii.edu (J.P.); dengy@hawaii.edu (Y.D.)

[†] These authors contributed equally to this work.

This file includes:

Figure S1-S4

Table S1.

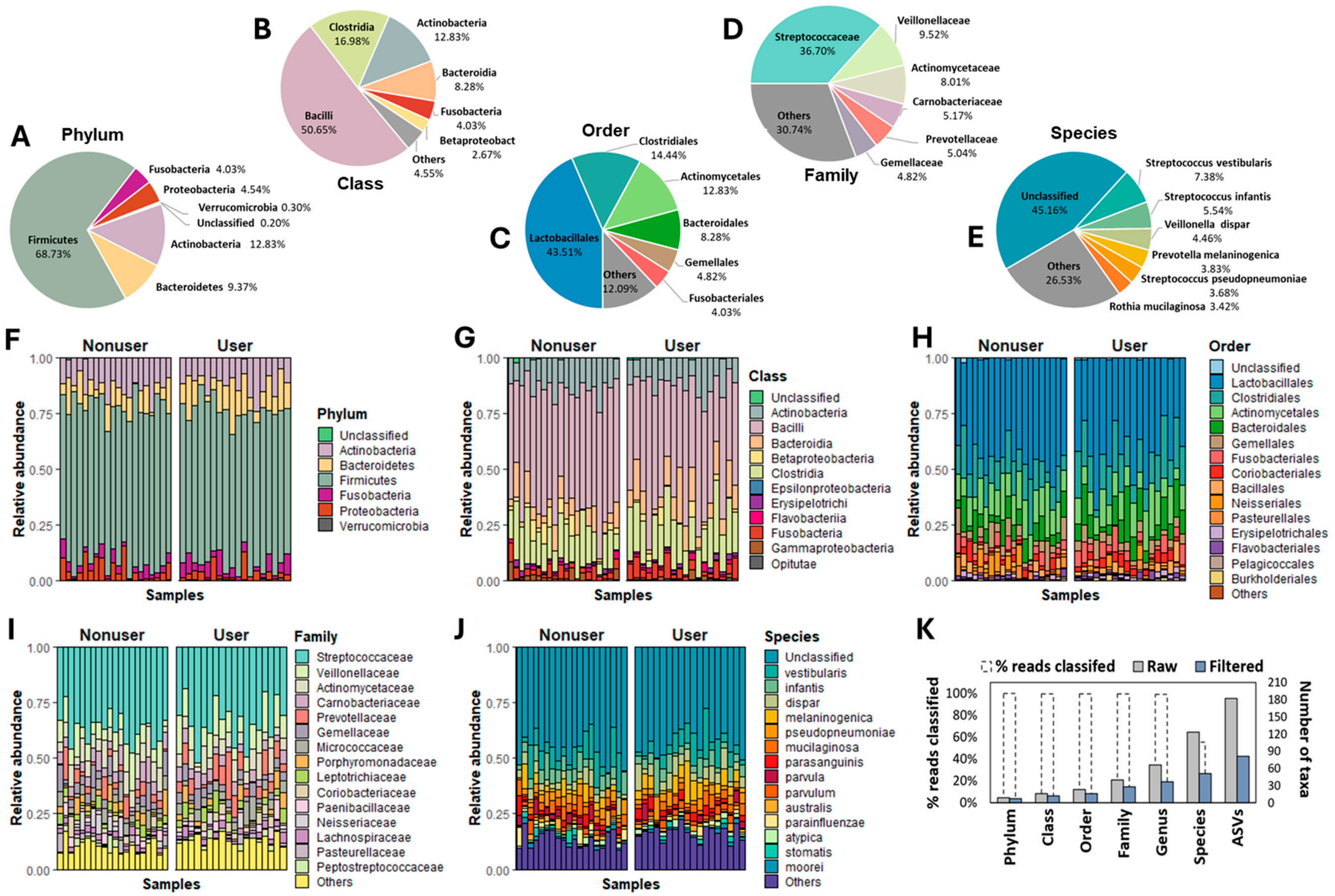

Figure S1. Overview of salivary microbiome composition at different taxonomic levels. (A-E) The top abundant taxa identified in the salivary microbiome in this study, at phylum, class, order, family and species level. (F-J) The top abundant taxa (<=15) detected in each salivary sample in both MJ user and non-users at different taxonomic levels. (K) Sequence taxonomic coverage (dash-outlined), number of taxa detected at each taxonomic level before (raw) and after removing singlets (filtered).

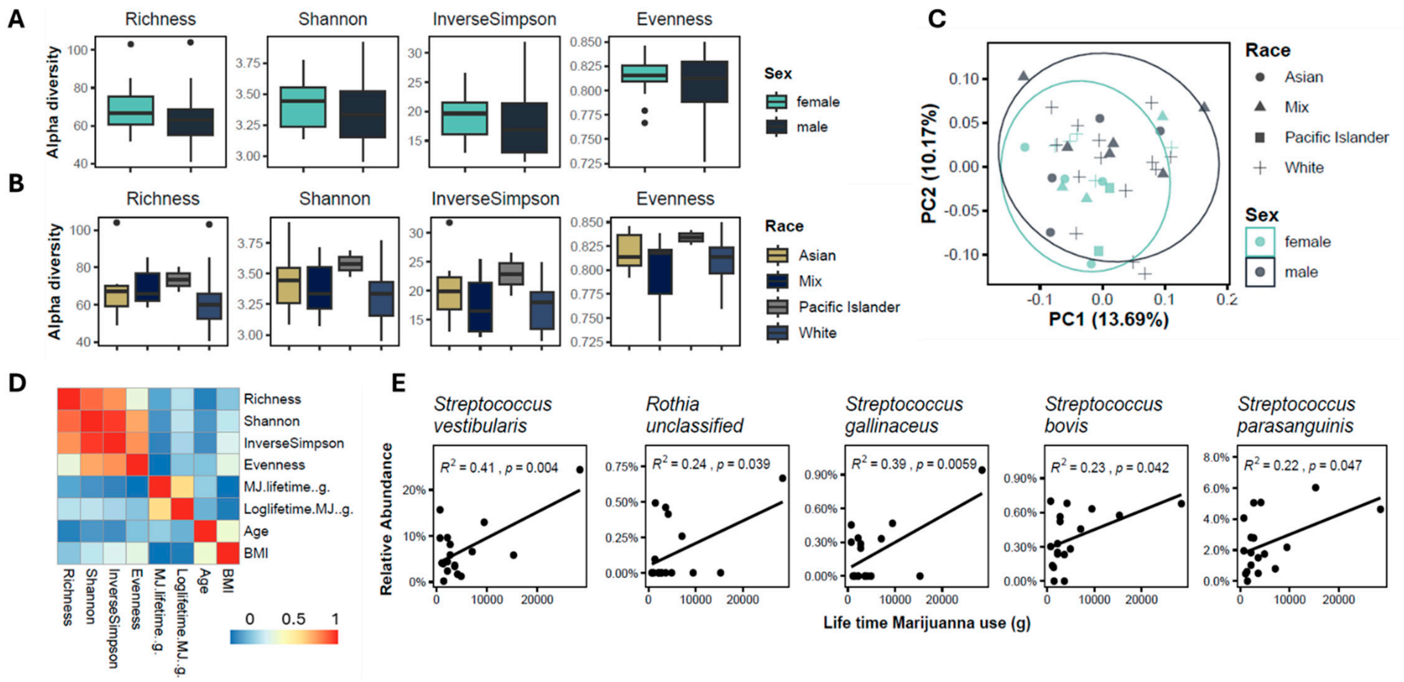

Figure S2. Salivary microbiome structures based on other demographic factors and species associated with lifetime MJ usage. (A-C) Salivary microbial community α-diversities based on sex (A) and race (B) and β-diversities (C) based on these groups. (D) The α-diversity indices and their correlations with accumulative lifetime MJ use, ages and BMIs in the MJ user group. (E) Linear regression shows the five salivary species have significant positive correlations between their relative abundances and the lifetime MJ use.

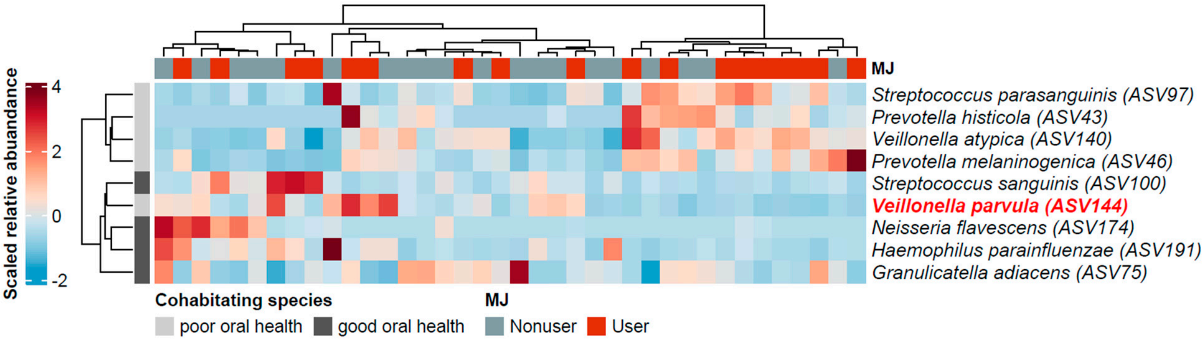

Figure S3. Clustering based on 9 salivary cohabitating species associated with good or poor oral health. *Veillonella parvula* did not fit into the oral health clustering and was not included in the cohabitating species for downstream clustering in this study.

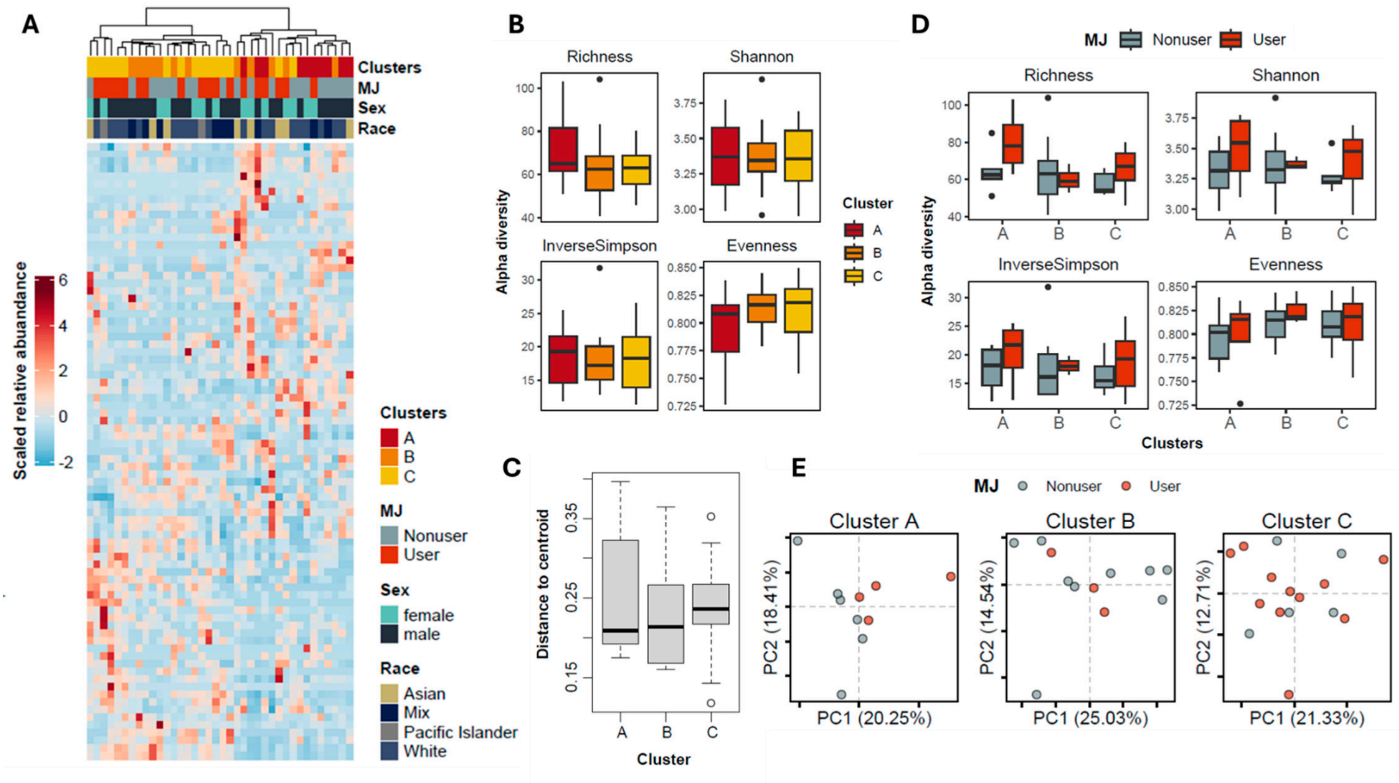

Figure S4. Microbial community analysis based on the three clusters (Cluster A, B, C) identified in Figure 2. (A) Amplicon sequence variant (ASV)-based microbial community clustering and inter-cluster comparisons. Two clusters were formed based on the relative abundance of all 81 ASVs identified in this study, notably Cluster A clearly grouped together again. (B) Overall α-diversity indices in each cluster (C) The dispersion levels of microbial communities in the three clusters, defined by the average distances to the cluster centroid. (D) Comparisons of α-diversity indices in each cluster between MJ user and non-users. (E) PCA plots to visualize the distances between salivary microbiomes in each cluster, the microbiomes are colored based on the MJ usage.

Table S1. Dissimilarity tests of saliva microbial community structure based on all 81 amplicon sequence variants (ASVs) identified in this study.

		ADONIS		ANOSIM		MRPP	
		R^2	*p*	R	*p*	Delta	*p*
**Marijuana**	User vs. nonusers	0.0378	0.166	0.0326	0.147	0.3847	0.163
**Sex**	Female vs. male	0.0321	0.283	0.0161	0.359	0.3857	0.269
**Race**	All race groups	0.0922	0.241	-0.0028	0.487	0.3834	0.249
**Clusters**	All clusters	0.1996	**0.001*** **	0.2822	**0.001*** **	0.3703	**0.001*** **
	Cluster A vs. B	0.0974	**0.021* **	0.1695	**0.02* **	0.3691	**0.011* **
	Cluster A vs. C	0.2183	**0.001*** **	0.5229	**0.001*** **	0.3784	**0.001*** **
	Cluster B vs. C	0.1277	**0.002** **	0.1651	**0.006** **	0.3636	**0.001*** **

* $p \leq 0.05$, ** $p \leq 0.01$, ***$p \leq 0.001$
